# Recommendations for Providing Quality Sexually Transmitted Diseases Clinical Services, 2020

**DOI:** 10.15585/mmwr.rr6805a1

**Published:** 2020-01-03

**Authors:** Roxanne Y. Barrow, Faruque Ahmed, Gail A. Bolan, Kimberly A. Workowski

**Affiliations:** ^1^Division of STD Prevention, National Center for HIV/AIDS, Viral Hepatitis, STD, and TB Prevention, CDC; ^2^Emory University, Atlanta, Georgia

## Abstract

This report (hereafter referred to as *STD QCS*) provides CDC recommendations to U.S. health care providers regarding quality clinical services for sexually transmitted diseases (STDs) for primary care and STD specialty care settings. These recommendations complement CDC’s *Sexually Transmitted Diseases Treatment Guidelines, 2015* (hereafter referred to as the *STD Guidelines*)*,* a comprehensive, evidence-based reference for prevention, diagnosis, and treatment of STDs. *STD QCS* differs from the *STD Guidelines* by specifying operational determinants of quality services in different types of clinical settings, describing on-site treatment and partner services, and indicating when STD-related conditions should be managed through consultation with or referral to a specialist. These recommendations might also help in the development of clinic-level policies (e.g., standing orders, express visits, specimen panels, and reflex testing) that can facilitate implementation of the *STD* *Guidelines*. CDC organized the recommendations for *STD QCS* into eight sections: 1) sexual history and physical examination, 2) prevention, 3) screening, 4) partner services, 5) evaluation of STD-related conditions, 6) laboratory, 7) treatment, and 8) referral to a specialist for complex STD or STD-related conditions.

CDC developed the recommendations by synthesizing relevant, evidence-based guidelines and recommendations issued by other experts; reviewing current practice in the United States; soliciting Delphi ratings by subject matter experts on STD care in primary care and STD specialty care settings; discussing the scientific evidence supporting the proposed recommendations at a consultation meeting of experts and institutional stakeholders held November 20, 2015, in Atlanta, Georgia; conducting peer reviews of draft recommendations and supporting evidence; and discussing draft recommendations and supporting evidence during meetings of the CDC/Health Resources and Services Administration Advisory Committee on HIV, Viral Hepatitis, and STD Prevention and Treatment STD Work Group. These recommendations are intended to help health care providers in primary care or STD specialty care settings offer STD services at their clinical settings and to help the persons seeking care live safer, healthier lives by preventing and treating STDs and related complications.

## Introduction

### Background

Approximately 20 million new cases of sexually transmitted diseases (STDs) occur every year in the United States, with approximately half occurring among persons aged 15–24 years (*1*). In recent years, STDs rates have increased (*2*). STDs account for $16.9 billion annually in health care costs (*3*). STDs can lead to severe reproductive health complications, such as infertility, ectopic pregnancy, and congenital infection. In addition, STDs can increase a person’s risk for acquiring and transmitting human immunodeficiency virus (HIV) infection (*4**,**5*).

STDs increasingly are being diagnosed in various health care settings. Most reported STD cases are from providers in non-STD clinics, such as private physician offices and community health centers (*2*). Historically, STDs were diagnosed in public health clinics for reasons of anonymity, confidentiality, and specialized care. A principle of STD care is timely management of infections, evidenced by the Brussels Agreement of 1924, an international treaty that sought to establish STD care in ports for merchant marines (*6*). In the United States, clinics dedicated to caring for patients with STDs, such as the first STD clinic in Baltimore, Maryland, which opened in 1922, offered confidential care to counteract the stigma of syphilis (*7*). These types of clinics increased in number during the 1930s and 1940s, and clinics have remained a large component of public health services (*8*). The framework for these STD clinics included timely diagnosis, testing with on-site treatment, and partner services.

During the 1980s and 1990s, most specialized STD care was provided in STD clinics and HIV programs (*9*). For patients, STD clinics were unique because they provided confidential, walk-in, low-cost specialty care (*9*) and offered the expertise necessary to manage STDs (*10*). However, because of funding issues, public health services and the number of STD clinics were reduced substantially during 2008–2012. Approximately half of local health departments reported reduction or elimination of at least one program, such as clinical health services or communicable disease screening and treatment, because of funding (*11*) and at least 10% of STD clinics closed (*12*).

Over time and with decreased availability of STD clinics, patients have sought care for STDs at primary care clinics, emergency departments, and family planning clinics (*13*). Primary care providers are an important component of sexual health care because many patients with STDs are asymptomatic and their infections might be identified while receiving services in the primary care setting. Certain studies have found that primary care clinics might diagnose up to half of reported STDs (*13*). In 2018, 71%–80% of STD cases were reported from non-STD clinics (*5*). One study that examined patients’ choice of providers for STD care found that, with expanded health care insurance coverage, patient visits to primary care providers increased more than 100% and STD clinic visits decreased 20%. This increase in primary care visits was largely attributable to a rise in the percentage of women seen for STD care (*14*). Despite these shifts in settings for STD service provision, publicly funded STD clinics continue to serve as a safety net for patients without insurance coverage or other marginalized groups of patients seeking care (*15*).

### Rationale

With increasing rates of most STDs in recent years (*2*), all providers have a role in the assessment of STD risk and management of infections. STD clinics will continue to be locations of expert care and are increasingly recognized as venues to provide HIV preexposure prophylaxis (PrEP) to prevent incident HIV infections (*16*). Providers in primary care offices, family planning clinics, and community-based clinics will continue to diagnose STDs among asymptomatic patients who are especially at risk for STDs. Recommendations for operationalizing STD care in health care settings are needed because provision of STD services varies. This report (hereafter referred to as *STD QCS*) describes what constitutes quality STD clinical services in primary care and STD specialty care settings.

In this report, provision of STD care is described as basic or specialized. Basic STD care usually is provided in primary care settings where patients are seen for various health conditions. Typically, specialized STD care is delivered in STD specialty care settings that focus on providing timely, comprehensive, confidential, and culturally sensitive STD care. Patients with STD-related conditions beyond the scope of both primary care and STD specialty care settings, such as those needing advanced diagnostics (e.g., lumbar punctures or ocular evaluations) or inpatient care, should be managed through consultation with or referral to a specialist.

CDC’s *Sexually Transmitted Diseases Treatment Guidelines, 2015* (hereafter referred to as the *STD Guidelines*) provides clinical guidance to physicians and other health care providers on the prevention, diagnosis, and treatment of STDs in the United States (*17*). The recommendations in *STD QCS* are intended to complement the *STD* *Guidelines*; as such, the *STD Guidelines* has not been modified. Rather, this report provides guidance on clinical operations and the types of services that should be available for STD care. *STD QCS* describes optimal services for the provision of quality STD-related clinical care by setting, including services that should be available at the time of the patient visit. Availability of same-day, on-site tests can reduce diagnostic delays and decrease excessive and costly presumptive treatment (*18*). On-site medications for STDs can minimize the duration of infectiousness and reduce STD transmission, decrease the cost of staff needed to follow up on positive tests and verify treatment, and lessen complications in the interval between testing and return visits for therapy (*19**,**20*). In settings where patient return rates are inconsistent, same-day services might result in more cases being diagnosed and more patients receiving timely treatment (*21*). Same-day treatment for patients and their sex partners is also critical for STD prevention and control because it can reduce transmission of STDs in the community.

In the Institute of Medicine* report *Crossing the Quality Chasm: A New Health System for the 21st Century,* the framework for health care quality was outlined using the following six domains: 1) safety, 2) effectiveness, 3) patient centeredness, 4) timeliness, 5) efficiency, and 6) equity (*22*). These domains are essential for the provision of quality STD clinical care services:

1. Safety: Patient safety includes the physical health care environment; seamless clinic processes; knowledge of the patients’ care plan; confidential services, especially confidential partner services; and an informed patient (*22**,**23*).
2. Effectiveness: Effective care includes providing services that are consistent with recognized medical and laboratory guidelines.
3. Patient centeredness: A patient-centered approach ensures
  - patient confidentiality,
  - attention to issues that disproportionally affect vulnerable populations,
  - guaranteeing a friendly and welcoming environment through cultural sensitivity,
  - a seamless referral process between health care providers, and
  - delivery of information and education relevant to a patient’s diagnosis, treatment, prognosis, and prevention measures.
4. Timeliness: Key factors in ensuring that services are provided in a timely manner include availability, accessibility, acceptability, and visibility of services (e.g., facility location, hours of operation, waiting time until appointment, waiting time in the clinic, staffing levels, and facility space); availability and selection of laboratory technologies and turnaround times for laboratory procedures; availability, accessibility, and cost of treatments; and availability of partner management and other prevention services, such as condoms and behavioral counseling (*18**,**23*). Timely and appropriate STD management can be influenced by several factors, including
  - patient health care–seeking behaviors,
  - diagnostic capabilities,
  - treatment capabilities, and
  - prevention activities.
5. Efficiency: An efficient health care system optimizes its resources by improving safety, effectiveness, patient centeredness, and timeliness.
6. Equity: Equity in health care ensures universal access to quality care.

### Scope and Audience

The recommendations in *STD QCS* apply to private and public providers of STD clinical services, including those in primary care settings (e.g., internal medicine, family medicine, or obstetrics-gynecology private offices; school-based health or community health centers; correctional health care settings; or HIV-care clinics) as well as those in sites dedicated to STD service delivery (e.g., STD or sexual health clinics). The focus is on structural-level policy recommendations about which STD-related clinical services should be available to facilitate implementation of the *STD* *Guidelines*.

In addition to these providers, *STD QCS* can also be used by others, such as

- medical directors to develop protocols that outline clinic procedures for delivering STD care,
- public health officials to establish partnerships with local care providers to reduce STD clinical service gaps,
- community organizations to stay informed about expected STD services for clients,
- health care administrators to measure or monitor health care facilities’ adherence to national recommendations,
- health care professionals and patients to advocate for quality services, and
- health care organizations to develop quality measures for STD services.

*STD QCS* recommendations address the following questions:

- What STD-related clinical services should be available to persons who have or are at risk for STDs, including asymptomatic persons, in primary care settings?
- What STD-related clinical services should be available to persons who have or are at risk for STDs in STD specialty care settings?
- Which STD-related conditions should be managed through consultation with or referral to a specialist?

These recommendations allow health care providers to build, maintain, or enhance the delivery of STD services in their primary care and STD specialty care settings. *STD QCS* is not intended to develop new guidance for when or how to provide the services or to mandate or regulate services. Health care settings might not provide every service outlined for quality STD care; however, the recommendations can provide the opportunity to assess which services are available in a facility and determine whether additional services can or should be made available or whether mechanisms for referral can or should be developed.

## Methods

### Overview

CDC developed these recommendations after consultation with a wide range of experts and stakeholders. CDC took into account existing national guidelines and recommendations, current practice in the United States, Delphi ratings by subject matter experts (SMEs) on STD care in primary care and STD specialty care settings followed by discussion at a consultation meeting, input of external private providers, and feedback from the CDC/Health Resources and Services Administration (HRSA) Advisory Committee on HIV, Viral Hepatitis, and STD Prevention and Treatment (CHAC).

In January 2015, CDC formed a steering committee that defined the scope of the proposed recommendations and provided feedback to CDC on the development process. SMEs on STD care in primary care and STD specialty care settings met via conference calls during June–September 2015 to provide individual feedback to CDC on the proposed recommendations. CDC developed draft recommendations that were discussed at a consultation meeting held November 20, 2015, in Atlanta, Georgia. The meeting included members of the steering committee, SMEs, federal agencies (i.e., CDC, Office of Population Health, and HRSA), and other stakeholders. Participants at the consultation meeting gave individual feedback on these draft recommendations. CDC sought additional input on the draft recommendations from private providers identified by stakeholders representing professional organizations (i.e., American Academy of Family Physicians [AAFP], American Academy of Pediatrics [AAP], American Congress of Obstetricians and Gynecologists [ACOG], and American College of Physicians [ACP]). Four private providers from AAFP provided input. Proposed revisions from CHAC were presented and approved at the October 2017 CHAC meeting in Rockville, Maryland.

All CDC staff, steering committee members, SMEs, and consultation meeting participants disclosed potential competing interests. CHAC and CHAC STD Work Group members also disclosed potential competing interests.

### Literature Review

Recommended STD-related clinical services were identified by reviewing relevant evidence-based guidelines and recommendations, including recommendations from the *STD* *Guidelines*, the Advisory Committee on Immunization Practices (ACIP), AAP, ACOG, the British Association for Sexual Health and HIV, the World Health Organization, and the U.S. Preventive Services Task Force (Supplementary Appendix 1, https://stacks.cdc.gov/view/cdc/82088). A systematic literature search also was conducted to identify current practice for STD screening in the United States (Supplementary Appendix 2, https://stacks.cdc.gov/view/cdc/82088). Medline and PsycInfo databases were searched for studies published from January 1, 1985, through May 26, 2015, and the *Sexually Transmitted Diseases* journal was searched from January 2006 through October 2015. Articles were screened using titles, and 414 abstracts and relevant full-text articles were retrieved. The reference lists of included studies were reviewed and the SMEs provided additional relevant citations (Supplementary Appendix 3, https://stacks.cdc.gov/view/cdc/82088). The inclusion criteria were articles describing original studies or systematic reviews that were published in English and that included U.S. populations or settings. Commentaries, letters, and editorials were excluded.

### Delphi Process

Using a modified Delphi process (a method that solicits the opinions of experts through a series of questionnaires), participating SMEs gave input on what clinical services should be available as basic and specialized STD care services (*24*). An online Delphi rating form, developed by CDC using SurveyMonkey, included definitions or indications for the clinical services. Discussions of the Delphi ratings were conducted (two rounds by the SMEs on STD care in primary care settings and three rounds by the SMEs on STD care in STD specialty care settings) with structured conference calls led by a moderator to obtain comments. The Delphi forms were modified on the basis of discussions during the conference calls. Aggregate results and comments were reviewed and discussed during the conference calls. Nine members of the primary care setting panel and nine members of the STD specialty care setting panel completed the ratings. The most important criteria for rating the clinical services was the association with quality of STD care and the feasibility of having a service available. SME panel members rated each clinical service on the basis of their clinical experience. Each service was rated using a scale from one to nine, where one indicated disagreement with the statement and nine indicated agreement. Median and dispersion of the ratings were analyzed to prioritize the clinical services, which were classified as appropriate (median rating of 7–9 without disagreement), inappropriate (median rating of 1–3 without disagreement), or uncertain (median rating of 4–6, or any median with disagreement). Disagreement was defined as at least three of the nine panelists rating a service outside the three-point region containing the median. A summary of the rating results is available (Supplementary Appendix 4, https://stacks.cdc.gov/view/cdc/82088).

### Federal Advisory Committee Review and Findings

CHAC is a federal advisory committee that advises the U.S. Department of Health and Human Services secretary, CDC director, and HRSA administrator about objectives, strategies, policies, and priorities for HIV, viral hepatitis, and STD prevention and treatment efforts. At the May 2017 CHAC meeting, the committee approved establishment of the CHAC STD Work Group to review and provide feedback on the CDC draft recommendations. The CHAC STD Work Group consisted of 17 SMEs from the public and private sectors. The work group met four times by telephone during August–September 2017 to review and discuss proposed revisions from individual members. The summary report was presented and approved at the October 2017 CHAC meeting. CHAC submitted a letter to the CDC director and the HRSA administrator outlining their findings.

### Recommendation Format

Recommendations are presented as described in the Grading of Recommendations Assessment, Development, and Evaluation (GRADE) (*25*). Strong recommendations are worded as “should” and weak recommendations are worded as “could.” A strong recommendation implies that all or almost all informed providers would choose the recommended course of action. A weak recommendation indicates that most informed providers would choose the recommended course of action but some would not. Delphi ratings were used to guide the development of recommendations, and clinical services reviewed were classified as appropriate, uncertain, or inappropriate and were worded as “should,” “could,” or “would not be expected,” respectively.

Current practice, discussion at the consultation meeting, and CHAC findings also were considered in formulating the strength of the recommendations. The specific guidance for prevention strategies, screening, diagnosis, and treatment are discussed briefly. More comprehensive information is available in the *STD Guidelines* and other references therein.

## Current Practice on Selected Clinical Services in the United States

STDs can be prevented using various strategies, including male latex condoms, behavioral counseling, preexposure vaccination, and presumptive treatment after exposure. Timely and appropriate treatment of persons with STDs is critical for reducing transmission and preventing complications. Prevention and timely treatment of STDs depend on several factors, including taking a sexual history, assessing risk for STDs, performing screening and diagnostic testing, providing on-site medications, and notifying and managing sex partners (*18*).

Obtaining a sexual history and assessing risk for STDs include the five Ps (i.e., partners, practices, protection, past STDs, and prevention of pregnancy) (*17*). Most primary care providers obtain a sexual history if it is relevant to the chief complaint but less frequently at an initial or routine annual visit and seldom at acute care, non-STD–related visits (*26**–**32*). Adolescent medicine clinicians, pediatricians, and obstetricians-gynecologists are more likely to regularly take a sexual history than family medicine clinicians, general practitioners, and internists (*26**,**28**,**29**,**33*). Primary care providers most commonly inquire about sexual activity and history of STDs but less often ask about the number of sex partners, gender of sex partners, and specific sexual practices (oral, vaginal, or anal sex) (*27**,**29**,**34**,**35*). Several studies have determined that STD clinics obtain more complete sexual histories than primary care clinics (*36**,**37*).

Screening and diagnostic testing are important to detect asymptomatic or confirm suspected infections. Previous studies have documented that one third to one half of primary care clinicians routinely screen men or women for STDs (chlamydia, gonorrhea, syphilis, or HIV) (*26**,**33**,**38**,**39*). Obstetricians-gynecologists screen nonpregnant women more often than other primary care physicians (*39**–**41*). Community health centers often provide routine HIV testing for pregnant women but less frequently offer routine HIV testing for men and nonpregnant women (*42**,**43*). Some emergency departments perform routine HIV testing, although the practice is not widespread (*44*). For correctional settings, routine testing varies by type of facility. State and federal prison systems commonly perform routine syphilis testing at intake but less often offer routine testing for HIV, gonorrhea, or chlamydia (*45*). Some city and county prisons conduct routine testing for syphilis but rarely offer routine testing for HIV, gonorrhea, or chlamydia (*45*). Jails and juvenile detention centers infrequently offer routine testing for STDs and rarely perform routine HIV testing (*46**,**47*).

In primary care clinics, Gram stain testing for gonorrhea usually is not performed on site because of difficulty obtaining Clinical Laboratory Improvement Amendments (CLIA) certification (*48*). Among 15 publicly funded STD clinics participating in the STD Surveillance Network, testing for *Trichomonas vaginalis* rarely involved culture, rapid antigen testing, or nucleic acid amplification test (NAAT); testing was almost exclusively performed using wet mount microscopy (*49*). A national survey of public health laboratories reported that only one fourth performed direct detection for syphilis. However, most laboratories conducted serological testing for syphilis (*50*). A survey of laboratories in the District of Columbia that reported gonorrhea results to the health department demonstrated that among laboratories that conducted gonorrhea cultures, only one third performed antibiotic susceptibility testing (*51*). A study of clinical laboratories in California reported that gonorrhea culture testing has substantially decreased and gonorrhea and chlamydia tests performed using NAAT have increased over time (*52*). For detection of herpes simplex virus (HSV), direct antigen and culture testing on lesions have decreased over time and serologic testing has increased (*52*).

If an infected patient receives timely treatment at an initial visit, further transmission can be interrupted. In one study, uninsured young adults were three to four times more likely to not fill a prescription (*53*). A study of primary and secondary syphilis cases reported to the Arizona STD Control Program indicated that treatment at the initial visit occurred more frequently for STD clinic patients compared with non-STD clinic patients (57% versus 8%) (*54**,**55*). A study of gonorrhea cases reported to the Philadelphia Department of Public Health indicated that among the 3,279 cases with documented treatment, 44% of the patients received same-day treatment. The median time to treatment for patients not treated the same day was 8 days (*56*). Among 1,241 patients with positive gonorrhea or chlamydia screening cultures who did not receive empiric treatment at their initial visit at an STD clinic in Alabama, 251 (20%) did not return to the clinic for treatment within 30 days of screening (*57*). Among 165 female patients with positive chlamydia tests who did not receive empiric treatment at their initial visit at a university medical center in Alabama, 55 (33%) emergency department or walk-in clinic patients and 14 (8%) obstetrics-gynecology patients had no treatment documented (*58*). A study of insured patients in 50 states indicated that 10% of new prescriptions for STD antimicrobials were not filled (*59*).

Notifying and treating sex partners interrupts transmission, prevents reinfection, and might prevent complications from unrecognized infections. Studies demonstrate that most primary care clinicians instruct patients with STDs to notify their sex partners for evaluation and treatment (*39**,**60**–**63*). Certain studies have found that approximately 50% of primary care clinicians reported ever having used expedited partner therapy (EPT) for chlamydia or gonorrhea but a substantially lower proportion (6%–27%) reported always or usually using this practice (*61**,**62**,**64**,**65*). Obstetricians-gynecologists and family medicine physicians reported using EPT more often than internal medicine physicians (*63**,**64*). A survey of community health centers in Indiana reported that 61% told patients with gonorrhea or chlamydia to refer their partner for testing and treatment and 18% always gave medication to patients to distribute to their partners (*66*). A survey of Federally Qualified Health Centers in New York City reported that 80% provided EPT for chlamydia, of which 47% were by prescription only, 27% by both prescription and dispensed medication, and 6% by dispensed medication only (*67*). A survey of family planning clinics in California found that 19% of partners received patient-delivered partner therapy (PDPT) and 55% received a patient referral. However, report of the partner receiving the treatment was higher for PDPT (80%) than for patient referral (44%) (*68**,**69*).

## Recommendations

*STD QCS* recommendations are outlined in the following eight sections: 1) sexual history and physical examination, 2) prevention, 3) screening, 4) partner services, 5) evaluation of STD-related conditions, 6) laboratory, 7) treatment, and 8) referral to a specialist for complex STD or STD-related conditions. Boxes 1–7 include the recommendations for basic STD care and specialized STD care. Citations of official guidelines and studies that support these recommendations accompany each section of recommendations. Box 8 includes a list of complex STD or STD-related conditions that primary care and STD specialty care settings should refer to a specialist.

### Sexual History and Physical Examination

A sexual history and risk assessment are foundational to providing quality STD care services (*17**,**70**,**71*). A complete sexual history includes inquiring about the five Ps (i.e., partners, practices, protection, past history of STDs, and prevention of pregnancy) (*17*). *A Guide to Taking a Sexual History* is available (https://www.cdc.gov/std/treatment/sexualhistory.pdf). The sexual history and risk assessment should be available as part of an initial comprehensive or annual visit; a visit for reproductive, genital, or urologic issues; or a visit for STD-related symptoms, STD-related concerns, or concerns about preventing or achieving pregnancy. The sexual history and risk assessment might be provided during an HIV, PrEP, or acute care visit.

A physical examination for STDs includes inspection of the skin, pharynx, lymph nodes, anogenital area, and neurologic system. The examination can provide useful information among males and females with STD-related symptoms (*17**,**70**–**73*). A physical examination allows health care providers the opportunity to identify the presence of any signs of STDs of which a patient might or might not be aware.

An anogenital examination for females includes a pelvic examination with three elements: 1) inspection of the external genitalia, urethral meatus, vaginal introitus, and perianal region; 2) speculum examination of the vagina and cervix; and 3) bimanual examination of the uterus, cervix, and adnexa (*72*). A colposcopy might be a useful procedure to examine the cervix, vagina, and vulva more closely for signs of disease and is recommended for those patients with abnormal Papanicolaou smear tests (Pap smears) according to the American Society for Colposcopy and Cervical Pathology (https://www.ncbi.nlm.nih.gov/pmc/articles/PMC3801360). An anogenital examination for males includes an external genital examination and inspection of the penis, scrotum, scrotal contents, and perianal region (*74*). An anoscopy can assist with the visualization of the anal canal among patients with rectal symptoms or history of receptive anal intercourse.

Sexual history and physical examination recommendations are summarized (Box 1). Additional information on sexual history and physical examination recommendations is available (Supplementary Appendix 1, https://stacks.cdc.gov/view/cdc/82088).

BOX 1 Recommendations for obtaining a sexual history and conducting a physical examination as part of sexually transmitted diseases care in primary care and sexually transmitted diseases specialty care settings*

**Table:** 

**STD care in primary care settings** A sexual history and risk assessment **should** be available as part of basic STD care services at the following patient visits: º Initial comprehensive or annual visit º Each visit concerning reproductive, genital, or urologic issues A physical examination **should** be available as a basic STD care service for male and female patients with STD-related symptoms, STD-related concerns, or those at high behavioral risk for incident STDs. A pelvic examination **should** be available as a basic STD care service. A sexual history and risk assessment **could** be available as basic STD care services at each visit unrelated to reproductive, genital, or urologic concerns. Anoscopy **could** be available as a basic STD care service.	**STD care in STD specialty care settings** A sexual history and risk assessment **should** be part of specialized STD care services at every visit for patients with STD-related symptoms, STD-related concerns, or concerns about preventing or achieving pregnancy. A physical examination **should** be available as a specialized STD care service for male and female patients with STD-related symptoms, STD-related concerns, or high behavioral risk for incident STDs. A pelvic examination **should** be available as a specialized STD care service. Colposcopy **should** be available as a specialized STD care service for female patients with abnormal Pap smears. Anoscopy **should** be available as a specialized STD care service. A high-resolution anoscopy **could** be available as a specialized STD care service for patients with abnormal anal Pap smears.

**Abbreviations:** Pap = Papanicolaou; STD = sexually transmitted disease.

* Primary care setting is defined as a place where patients are evaluated for various health conditions. An STD specialty care setting is defined as a place where the focus is on providing patients with timely, comprehensive, confidential, and culturally sensitive STD care. STD care delivered in STD specialty care settings includes all care delivered in primary care settings.

### Prevention

Services for preventing STDs and related conditions, including HIV, consist of eight strategies. These are 1) condom provision (*17**,**70**,**75*); 2) hepatitis A vaccination (*17**,**76*); 3) hepatitis B vaccination (*17**,**77*); 4) human papillomavirus (HPV) vaccination (*78**,**79*); 5) emergency contraceptive pills (*17**,**80**,**81*); 6) STD/HIV prevention counseling (brief, moderate intensity, or high intensity) (*17**,**82**–**84*); 7) PrEP for HIV prevention risk assessment, education, counseling, provision, and linking or referral, or both (*85*); and 8) nonoccupational postexposure prophylaxis (nPEP) of HIV risk assessment, education, counseling, provision, and linking or referral, or both, to HIV care (*86*).

STD/HIV prevention counseling, including behavioral counseling methods, can be used by health care providers to effect patient changes to reduce the patient’s risk for STD acquisition and transmission. The methods vary in scope and time. Brief prevention counseling is conducted in a single session using strategies, such as motivational interviewing and building rapport, and includes patient circumstances and needs in the counseling plan. Moderate-intensity and high-intensity behavioral counseling is contact time of 30–120 minutes and ≥2 hours, respectively (*82*). Contraceptive counseling enables clients to make and follow through on decisions about their contraceptive use. Education is an integral component of the contraceptive counseling process and helps clients make informed decisions and obtain information needed to use contraceptive methods correctly. Guidance for clinical providers on contraceptive services are outlined in *Providing Quality Family Planning Services: Recommendations of CDC and the U.S. Office of Population Affairs* (*87*). Linkage to care and helping patients start HIV medical care shortly after diagnosis can enhance prompt delivery of important services and support efforts to eliminate HIV infection (*83*). Linkage to HIV care, family planning, and behavioral health services can increase the proportion of those living with HIV who are virally suppressed, reduce unintended pregnancies, and maximize long-term behavioral health. Guidance for clinical providers on linkage to and retention in HIV medical care is outlined in the *Recommendations for HIV Prevention with Adults and Adolescents with HIV in the United States, 2014 Summary for Clinical Providers* (*88*).

Prevention recommendations are outlined (Box 2). Additional information on prevention recommendations is available (Supplementary Appendix 1, https://stacks.cdc.gov/view/cdc/82088).

BOX 2 Prevention recommendations for sexually transmitted diseases care in primary care and sexually transmitted diseases specialty care settings*

**Table:** 

**STD care in primary care settings** The following prevention services **should** be available as basic STD care services: º On-site hepatitis B vaccination or referral º On-site HPV vaccination or referral º Brief single STD/HIV prevention counseling session (up to 30 minutes)^†^ º PrEP for HIV prevention and nPEP of HIV risk assessment, education, and referral or link to HIV care^†^ º Emergency contraceptive pills^§^ º Brief contraceptive counseling or referral º Referral or link to HIV care, family planning services, and behavioral health services, if indicated The following prevention services **could** be available as basic STD care services: º On-site condom provision^¶^ º On-site hepatitis A vaccination º Provision of PrEP for HIV prevention** º Provision of nPEP of HIV^††^ º Moderate-intensity STD behavioral counseling (≥30 minutes)^†^	**STD care in STD specialty care settings** The following prevention services **should** be available as specialized STD care services: º On-site condom provision º On-site hepatitis A vaccination º On-site hepatitis B vaccination º On-site HPV vaccination º Brief single STD/HIV prevention counseling session (up to 30 minutes)^†^ º PrEP for HIV prevention and nPEP of HIV risk assessment, education, counseling, and referral or link to HIV care^†^ º Provision of PrEP for HIV prevention^§§^ º Provision of nPEP of HIV^¶¶^ º Brief contraceptive counseling or referral º Emergency contraceptive pills^§^ º Referral or link to HIV care, family planning services, and behavioral health services, if indicated The following prevention services **could** be available as specialized STD care services: º Moderate-intensity STD behavioral counseling (≥30 minutes)^†^ º High-intensity STD behavioral counseling (≥2 hours)^†^

**Abbreviations:** HIV = human immunodeficiency virus; HPV = human papillomavirus; nPEP = nonoccupational postexposure prophylaxis; PrEP = preexposure prophylaxis; STD = sexually transmitted disease.

* Primary care setting is defined as a place where patients are evaluated for various health conditions. STD specialty care setting is defined as a place where the focus is on providing patients with timely, comprehensive, confidential, and culturally sensitive STD care. STD care delivered in STD specialty care settings includes all care delivered in primary care settings.

^†^ Provided by a clinician or other appropriately trained staff.

^§^ If emergency contraceptive pills are not available on site or by prescription, patients can be advised that levonorgestrel emergency contraceptive pills are available over the counter and ulipristal acetate emergency contraceptive pills are only available by prescription. Emergency contraceptive pills should be taken as soon as possible within 5 days of unprotected sex.

^¶^ Providers can partner with local organizations, such as the local health department and community-based organizations, to procure condoms. In some states, prescriptions can be written for condoms. For certain settings, such as family planning clinics, condoms should be available on site.

** PrEP could be available by starter packs or prescription with on-site follow-up care for basic STD care. If PrEP is not provided, navigator-assisted referral for PrEP should be provided with first appointment made while the patient is on site.

^††^ nPEP starter pack (3–7 days of medication) could be available on site, with either on-site follow-up care or referral for basic STD care. nPEP starter pack or complete 28-day course could be available by prescription, with either on-site follow-up care or referral, with first appointment made while the patient is on site. Provision of the complete 28-day nPEP medication supply at the initial visit rather than a starter pack of 3–7 days has been reported to increase likelihood of adherence, especially when patients find returning for multiple follow-up visits difficult. Routinely providing starter packs or the complete 28-day course requires that health care providers stock nPEP drugs in their practice setting or have an established agreement with a pharmacy to stock, package, and urgently dispense nPEP drugs with required administration instructions (https://www.cdc.gov/hiv/pdf/programresources/cdc-hiv-npep-guidelines.pdf).

^§§^ PrEP should be available in starter packs or by prescription with on-site follow-up care for specialized STD care. If PrEP is not provided, navigator-assisted referral for PrEP should be provided with first appointment made while the patient is on site.

^¶¶^ nPEP starter pack (3–7 days of medication) should be available on site, with either on-site follow-up care or referral to specialized STD care. nPEP complete 28-day course should be available by prescription, with either on-site follow-up care or referral, with first appointment made while the patient is on site. Provision of the complete 28-day nPEP medication supply at the initial visit rather than a starter pack of 3–7 days has been reported to increase likelihood of adherence, especially when patients find returning for multiple follow-up visits difficult.

### Screening

Screening for asymptomatic STDs is important for early detection and prevention of STDs. Because many STDs are asymptomatic, testing is the only method to diagnose these infections. The availability of screening tests are key for identifying gonorrhea, chlamydia, syphilis, hepatitis B, hepatitis C, HIV, trichomoniasis, and cervical and anal cancer (*17**,**84**,**89**–**99*). Results from these screening tests can be used to identify persons at risk for STDs (*17*). Data are insufficient to recommend routine anal cancer screening with anal cytology among persons with HIV infection, men who have sex with men (MSM) without HIV infection, and the general population (*17*). However, some clinical centers perform anal cytology to screen for anal cancer among high-risk populations followed by a high-resolution anoscopy for those with abnormal cytologic results. Colposcopy is a recommended tool for cervical cancer screening.

A table summarizing screening recommendations for women, pregnant women, men, MSM, and persons with HIV is available (https://www.cdc.gov/std/treatment-guidelines/). Recommendations for STD screening are listed (Box 3). Additional information on screening recommendations is available (Supplementary Appendix 1, https://stacks.cdc.gov/view/cdc/82088).

BOX 3 Screening recommendations for sexually transmitted diseases care in primary care and sexually transmitted diseases specialty care settings*

**Table:** 

**STD care in primary care settings** Screening and assessment for the following STDs **should** be available as basic STD care services: º Gonorrhea º Chlamydia º Syphilis º Hepatitis B º Hepatitis C º HIV º Cervical cancer Screening and assessment for the following STD **could** be available as a basic STD care service: º Trichomoniasis	**STD care in STD specialty care settings** Screening and assessment for the following STDs **should** be available as specialized STD care services: º Gonorrhea º Chlamydia º Syphilis º Hepatitis B º Hepatitis C º HIV º Cervical cancer º Trichomoniasis Screening and assessment for the following STD **could** be available as a specialized STD care service: º Anal cancer

**Abbreviations:** HIV = human immunodeficiency virus; STD = sexually transmitted disease.

* Primary care setting is defined as a place where patients are evaluated for various health conditions. STD specialty care setting is defined as a place where the focus is on providing patients with timely, comprehensive, confidential, and culturally sensitive STD care. STD care delivered in STD specialty care settings includes all care delivered in primary care settings.

### Partner Services

Treatment of sex partners prevents reinfection and is essential to interrupting transmission of STDs. Partner services consist of various strategies with differing levels of time and effort to enable persons who are exposed to an STD to be identified, tested, and treated. These strategies include 1) guidance regarding notification and care of sex partners, 2) interactive counseling for partner notification, 3) EPT, and 4) health department disease intervention specialist (DIS) elicitation of sex partner information to identify those who might be infected and to identify patient follow-up needs (*17**,**83**,**100**–**106*).

Guidance regarding notification and care of sex partners is described as providers giving how-to information to their patients about the need to notify their sex partner or partners of the exposure, the need for sex partners to seek care and treatment even if they do not have symptoms, and where a partner could go for STD care. This strategy typically does not require extensive staff training. Guidance might be verbal or written. When notifying patients of an STD diagnosis and need to return for treatment, providers can advise patients to bring their sex partner with them, at which time the provider should treat both persons concurrently (*69*).

In interactive counseling, the provider and patient both actively participate in an individualized plan to notify the patient’s sex partner or partners. Interactive counseling typically is conducted by staff with specific training or skills in communication, interviewing, or counseling. The patient provides information about their sex partner or partners and develops a plan with the counselor to notify partners. Notification might involve the patient, the provider, or the health department. Efforts to notify partners can be documented and assessed.

EPT typically is recommended for sex partners of patients who have received a diagnosis of chlamydia or gonorrhea, or both, and who are unlikely to access timely care. This is a method that provides medications or prescriptions to the patient to take to a partner without the partner first being examined by a health care provider. EPT is legislated or regulated at the state level, and what each state allows can vary by STD, age group, and sexual orientation. Details about EPT are available (https://www.cdc.gov/std/ept/default.htm). For these methods, treatment and infection status of the partner can be verified and documented along with any co-occurring conditions (e.g., HIV infection or pregnancy) and health care access.

A DIS is a public health professional with applied expertise in client-centered interviews; partner services that include contact tracing, directly observed therapy, field specimen collection, and field investigation in outbreaks; and navigation of health care systems to ensure patient evaluation and treatment, among other areas (*107*). The position does not require a license, although persons with medical licenses sometimes serve in DIS positions. Relevant program areas include STD, HIV, tuberculosis, other communicable diseases, outbreak investigation, and emergency preparedness and response. DISs investigate STDs mandated by jurisdictional needs. Providers in health care settings are encouraged to develop relationships with their local health departments so that they can involve DISs in the cases routinely investigated on the basis on resources available in the jurisdiction and better inform their patients about DIS services.

Partner services recommendations are outlined (Box 4). Additional information on these recommendations is available (Supplementary Appendix 1, https://stacks.cdc.gov/view/cdc/82088).

BOX 4 Partner services recommendations for sexually transmitted diseases care in primary care and sexually transmitted diseases specialty care settings*

**Table:** 

**STD care in primary care settings** The following partner services **should** be available as basic STD care services: º Guidance regarding notification and care of sex partners º EPT (where legal and where local or state jurisdictions do not prohibit by regulation)^†^ The following partner services **could** be available as a basic STD care service: º Interactive counseling for partner notification	**STD care in STD specialty care settings** The following partner services **should** be available as specialized STD care services: º Guidance regarding notification and care of sex partners º Interactive counseling for partner notification º EPT (where legal and where local or state jurisdictions do not prohibit by regulation)^†^ º Health department DIS elicitation of sex partner information to identify those who might have been exposed and to identify patient follow-up needs^§^

**Abbreviations:** DIS = disease intervention specialist; EPT = expedited partner therapy; STD = sexually transmitted disease.

* Primary care setting is defined as a place where patients are evaluated for various health conditions. STD specialty care setting is defined as a place where the focus is on providing patients with timely, comprehensive, confidential, and culturally sensitive STD care. STD care delivered in STD specialty care settings includes all care delivered in primary care settings.

^†^ Information on legal status of EPT for each state is available at https://www.cdc.gov/std/ept/legal/default.htm.

^§^ Partner services can be provided on site or by referral.

### Evaluation of STD-Related Conditions

Genital ulcer disease can be caused by syphilis, HSV, chancroid, granuloma inguinale, and lymphogranuloma venereum (LGV). The more common sexually transmitted causes of male urethritis syndrome can include gonorrhea, chlamydia, mycoplasma, trichomoniasis, and HSV. Diseases characterized by vaginal discharge as a result of vaginitis can include bacterial vaginosis, trichomoniasis, and candidiasis. The causes of epididymitis, pharyngitis, cervicitis, and pelvic inflammatory disease (PID) can include gonorrhea and chlamydia. Most genital warts are caused by nononcogenic HPV types. However, on occasion, oncogenic types can be found in genital warts. Proctitis can be caused by gonorrhea, LGV serovars of *Chlamydia trachomatis*, syphilis, and HSV. Ectoparasitic infections typically include pediculosis pubis and scabies. Systemic or dermatologic conditions compatible with or suggestive of an STD can include disseminated gonorrhea, neurosyphilis, ocular syphilis, condylomata lata, or palmar plantar syphilitic rash. STD-related conditions warrant prompt evaluation of signs and symptoms to make an accurate diagnosis and provide timely empiric treatment to prevent complications and onward transmission. Patients with a clinical presentation suggestive of an STD etiology (genital ulcer disease, urethritis, vaginal discharge, PID, epididymitis, pharyngitis, genital warts, proctitis, ectoparasitic infections, or certain systemic or dermatologic conditions) should be evaluated (*17**,**70**,**71*).

Recommendations for the STD-related conditions that should be evaluated are summarized (Box 5). Additional information on these recommendations is available (Supplementary Appendix 1, https://stacks.cdc.gov/view/cdc/82088).

BOX 5 Evaluation of sexually transmitted disease–related conditions recommendations in primary care and sexually transmitted diseases specialty care settings*

**Table:** 

**STD care in primary care settings** Evaluation (history and examination) for the following STD-related conditions **should** be available as basic STD care services: º Genital ulcer disease º Male urethritis syndrome º Vaginal discharge º PID º Genital warts º Proctitis^†^ º Ectoparasitic infections º Pharyngitis º Epididymitis º Systemic or dermatologic conditions compatible with or suggestive of an STD etiology	**STD care in STD specialty care settings** Evaluation (history and examination) for the following STD-related conditions **should** be available as specialized STD care services: º Genital ulcer disease º Male urethritis syndrome º Vaginal discharge º PID º Genital warts º Proctitis^†^ º Ectoparasitic infections º Pharyngitis º Epididymitis º Systemic or dermatologic conditions compatible with or suggestive of an STD etiology

**Abbreviations:** PID = pelvic inflammatory disease; STD = sexually transmitted disease.

* Primary care setting is defined as a place where patients are evaluated for various health conditions. STD specialty care setting is defined as a place where the focus is on providing patients with timely, comprehensive, confidential, and culturally sensitive STD care. STD care delivered in STD specialty care settings includes all care delivered in primary care settings.

^†^ Evaluation for proctitis might include visual examination of the anus, anorectal examination with a rectal swab, digital anorectal exam, or anoscopy. For specialized STD care, high-resolution anoscopy might be included.

### Laboratory

Tests cleared by the U.S. Food and Drug Administration (FDA) are available for identifying STDs. Certain tests can be performed as point-of-care tests, either on site or through a clinical laboratory with rapid turnaround times. Providing results during the same clinic visit is ideal. Typically, these tests are waived by CLIA or are moderately complex. Use of commercially available NAATs for gonorrhea and chlamydia is cleared by FDA for genital tract specimens (*17**,**90*).

Laboratory tests for identifying STDs are important for screening and diagnostic purposes. Screening tests are the only method for identifying asymptomatic infections. To improve rates of recommended screenings, primary care and STD specialty care providers can implement structural-level policy interventions in clinical settings, such as standing orders for the registration staff, express visits, specimen panels, and reflex testing. For patients, a sexual history and risk assessment can help determine whether a screening test is necessary. For patients with STD-related symptoms, both a physical examination and laboratory testing are needed along with a sexual history and risk assessment to determine the possible cause of symptoms and provide an opportunity for the diagnosis of other, unrecognized infections. In STD specialty care settings, same-day diagnosis and treatment are core functions for both health care and public health outcomes. With rapid laboratory results, treatment delays are reduced, resulting in fewer complications, less onward transmission of STDs, less time spent tracking and verifying treatment for those who fail to return after a positive test result, and more prudent use of antimicrobials based on less empiric treatment. STD specialty care settings should offer same-day diagnostic testing and treatment for patients with STD-related conditions and for sex partners of patients with a diagnosed sexually transmitted infection. Laboratory recommendations are outlined (Box 6).

BOX 6 Laboratory recommendations for sexually transmitted diseases care in primary care and sexually transmitted diseases specialty care settings*

**Table:** 

**STD care in primary care settings** ***At the time of the patient visit*** The following general services, equipment, or tests **should** be available as basic STD care services at the time of the patient visit: º Thermometer º pH paper The following general services, equipment, or tests **could** be available as basic STD services with test results available during the patient visit: º Phlebotomy º Test for trichomoniasis^†^ º Test for bacterial vaginosis^§^ º Test for vulvovaginal candidiasis^¶^ º Urine dipstick º Urinalysis with microscopy º Test for pregnancy º Test for HIV ***Clinical laboratory*** The following tests **should** be available through a clinical laboratory as basic STD care services: º Urogenital NAAT for gonorrhea and chlamydia º Extragenital (pharynx and rectum) NAAT for gonorrhea and chlamydia º Quantitative nontreponemal serologic test for syphilis º Treponemal serologic test for syphilis º HSV viral culture or PCR º HSV serology º Fourth-generation antigen/antibody HIV test º Oncogenic HPV NAATs with Pap smear º nPEP and PrEP º Serologic tests for hepatitis A, B, and C º Test for pregnancy The following tests **could** be available through a clinical laboratory as basic STD care services: º Gram stain, methylene blue, or gentian violet stain for urethritis º Gonorrhea culture º Gonorrhea antimicrobial susceptibility testing** º NAAT for trichomoniasis	**STD care in STD specialty care settings** ***At the time of the patient visit*** The following general services, equipment, or tests **should** be available as specialized STD care services at the time of the patient visit: º Thermometer º pH paper º Phlebotomy º Test for trichomoniasis^†^ º Test for bacterial vaginosis^§^ º Test for vulvovaginal candidiasis^¶^ º Urine dipstick º Urinalysis with microscopy º Test for pregnancy º Gram stain, methylene blue, or gentian violet stain for urethritis º On-site qualitative nontreponemal serologic test for syphilis The following general services, equipment, or tests **could** be available as specialized STD care services with test results available during the patient visit: º Dark-field microscopy for syphilis º Test for HIV ***Clinical laboratory*** The following tests **should** be available through a clinical laboratory as specialized STD care services: º Urogenital NAAT for gonorrhea and chlamydia º Extragenital (pharynx and rectum) NAAT for gonorrhea and chlamydia º Quantitative nontreponemal serologic test for syphilis º Treponemal serologic test for syphilis º HSV viral culture or PCR º HSV serology º Fourth-generation antigen/antibody HIV test º Oncogenic HPV NAATs with Pap smear º nPEP and PrEP º Serologic tests for hepatitis A, B, and C º Gonorrhea culture º Gonorrhea antimicrobial susceptibility testing** º NAAT for trichomoniasis

**Abbreviations:** HIV = human immunodeficiency virus; HPV = human papillomavirus; HSV = herpes simplex virus; NAAT = nucleic acid amplification test; nPEP = nonoccupational postexposure prophylaxis; Pap = Papanicolaou; PCR = polymerase chain reaction; PrEP = preexposure prophylaxis; STD = sexually transmitted disease.

* Primary care setting is defined as a place where patients are evaluated for various health conditions. STD specialty care setting is defined as a place where the focus is on providing patients with timely, comprehensive, confidential, and culturally sensitive STD care. STD care delivered in STD specialty care settings includes all care delivered in primary care settings.

^†^ On-site test for trichomoniasis can include wet mount microscopy and OSOM Trichomonas.

^§^ On-site test for bacterial vaginosis can include wet mount microscopy, OSOM BVBlue, and Affirm.

^¶^ On-site test for vulvovaginal candidiasis can include wet mount microscopy.

** Access needs to be established for transport medium that adequately maintains the viability of *Neisseria gonorrhoeae* until the specimen reaches a laboratory (e.g., transport medium in transport container, transport system, or transport swab). Providers should contact their state or local health department if they have concerns about resistant *N. gonorrhoeae* infection or if assistance is required for culture and antimicrobial susceptibility testing.

### Treatment

In settings where same-day treatment is available for patients with STD-related conditions and for sex partners of patients with a diagnosed STD, treatment should not be delayed while awaiting diagnostic test results. Delays in treatment might increase complications and contribute to transmission of infection in the community; and same-day treatment has numerous public health benefits. In an STD specialty care setting, same-day treatment should take place on site with the provision of a full course of appropriate medication. The first dose should be administered while the patient is in the clinic.

The *STD* *Guidelines* includes recommended regimens for treating STDs (*17*). An STD treatment guide app (STD Tx Guide), based on the *STD Guidelines*, is available free of charge for Apple and Android devices (https://www.cdc.gov/std/treatment-guidelines/). Wall charts and pocket guides that include a summary of the guidelines for recommended medications, doses, and duration of therapy are also available at the link.

For STD care in primary care settings, the following treatments could be available on site: recommended medications for chlamydia and gonorrhea, medications used as first-line therapies for STD-related conditions (urethritis, cervicitis, PID, epididymitis, and proctitis), recommended medications for syphilis, emergency contraceptive pills, PrEP, nPEP, and provider-applied regimens for genital warts. If medications are not available on site, they should be available by prescription. Ideally, tracking systems to verify that the medications were picked up should be established for STD prescriptions. Providers might not receive reimbursement for oral medications without an on-site pharmacy. Providers can partner with local organizations, such as the local health department and community-based organizations, to procure oral medications. For syphilis treatment, providers can partner with local health departments to procure injectable medication.

For STD care in STD specialty care settings, recommended medications for common STDs and related conditions should be available on site with the exception of medications for bacterial vaginosis, candida vaginitis, urinary tract infections, ectoparasitic infections, and patient-applied regimens for genital warts. If medications are not available on site, they should be available by prescription. Sex partners should be treated as outlined in the *STD Guidelines*. Medications for sex partners of patients with gonorrhea, or both, should also be available on site and managed in accordance with state EPT laws and regulations. Alternative medications for chlamydia, gonorrhea, and syphilis, provider-applied regimens for genital warts, emergency contraceptive pills, and nPEP should be available on site. PrEP could be available on site. Specific guidance on provision of nPEP starter packs or a 28-day supply at initiation is available (https://stacks.cdc.gov/view/cdc/38856). Information on linkage to care is available (https://stacks.cdc.gov/view/cdc/44065). The treatment recommendations are listed (Box 7).

BOX 7 Treatment recommendations for sexually transmitted diseases care in primary care and sexually transmitted diseases specialty care settings*

**Table:** 

**STD care in primary care settings** ***On site*** Medications for the following **could** be available on site as basic STD care services: º Gonorrhea^†^ º Chlamydia^†^ º Cervicitis º Nongonococcal urethritis^†^ º Proctitis º PID º Epididymitis º Syphilis^§^ º PrEP º nPEP º Provider-applied regimens for genital warts º Emergency contraceptive pills^¶^ ***Prescription*** All recommended medications for the following **should** be available by prescription as basic STD care services: º EPT for gonorrhea and chlamydia** º Herpes º Trichomoniasis º Bacterial vaginosis º Vulvovaginal candidiasis º UTI º PrEP º nPEP º Emergency contraceptive pills^¶^ º Patient-applied regimens for genital warts º Ectoparasitic infections	**STD care in STD specialty care settings** ***On site*** Medications for the following **should** be available on site as specialized STD care services: º Gonorrhea º Chlamydia º Cervicitis º Nongonococcal urethritis º Proctitis º PID º Epididymitis º Syphilis º nPEP º Provider-applied regimens for genital warts º Emergency contraceptive pills^¶^ º EPT for gonorrhea and chlamydia** º Herpes º Trichomoniasis Medications for the following **could** be available on site as specialized STD care services: º Bacterial vaginosis º Acute or new diagnosis of HIV care º PrEP º Persistent and recurrent cervicitis and nongonococcal urethritis ***Prescription*** All recommended medications for the following **should** be available by prescription as specialized STD care services: º Vulvovaginal candidiasis º UTI º PrEP º Patient-applied regimens for genital warts º Ectoparasitic infections

**Abbreviations:** EPT = expedited partner therapy; HIV = human immunodeficiency virus; nPEP = nonoccupational postexposure prophylaxis; PID = pelvic inflammatory disease; PrEP = preexposure prophylaxis; STD = sexually transmitted disease; UTI = urinary tract infection.

* Primary care setting is defined as a place where patients are evaluated for various health conditions. STD specialty care setting is defined as a place where the focus is on providing patients with timely, comprehensive, confidential and culturally sensitive STD care. STD care delivered in STD specialty care settings includes all care delivered in primary care settings.

^†^ Providers might not receive reimbursement for oral medications without an on-site pharmacy. Providers can partner with local organizations, such as the local health department and community-based organizations, to procure oral medications or refer patients to local organizations.

^§^ Providers can partner with local health departments to procure injectable benzathine penicillin G or refer patients to local health department and verify treatment.

^¶^ If emergency contraceptive pills are not available on site or by prescription, patients can be advised that levonorgestrel emergency contraceptive pills are available over the counter and ulipristal acetate emergency contraceptive pills are only available by prescription. Emergency contraceptive pills should be taken as soon as possible within 5 days of unprotected sex.

** Information on the legal status of EPT for each state is available at https://www.cdc.gov/std/ept/legal/default.htm.

### Referral to a Specialist for Complex STD or STD-Related Conditions

The *STD Guidelines* specifies conditions that should be managed through referral to a specialist (*17*). Referrals should be made to clinicians who have extensive specialized training or experience in diagnosing, treating, and providing follow up for complex STD cases. These providers can include adult and pediatric infectious disease clinicians, maternal-fetal medicine specialists, allergists, ophthalmologists, gastroenterologists, colorectal surgeons, urologists, oncologists, and other specialists. Services can be provided in different sites within a multispecialty practice or hospital system. Recommendations on referral to a specialist for complex STD or STD-related conditions are described (Box 8).

BOX 8 **Recommendations on referral to specialist for complex sexually transmitted diseases or sexually transmitted disease–related conditions**

**Complex gonorrhea**

- Antimicrobial-resistant gonorrhea
- Cephalosporin or IgE-mediated penicillin allergy
- Suspected cephalosporin treatment failure
- Gonococcal conjunctivitis
- Disseminated gonococcal infection or gonococcal endocarditis or meningitis
- Gonococcal ophthalmia in infants

**Complex chlamydial infections**

- Chlamydial ophthalmia in infants
- Pneumonia in infants
- Persistent or recurrent epididymitis
- Persistent or recurrent cervicitis
- Cephalosporin or IgE-mediated penicillin allergy
- Suspicion of testicular torsion

**Complex syphilis**

- Primary, secondary, and latent syphilis in infants and children
- IgE-mediated penicillin allergy
- Tertiary syphilis
- Neurosyphilis
- Ocular or otic syphilis
- Syphilis during pregnancy with sonographic signs of fetal or placental syphilis

**Complex vaginal discharge, trichomoniasis, and candidiasis**

- Persistent vaginal discharge of unclear etiology
- Persistent or recurrent trichomoniasis
- IgE-mediated allergy to nitroimidazoles
- Recurrent vulvovaginal candidiasis in patients who remain culture-positive despite maintenance therapy
- Recurrent nonalbicans vulvovaginal candidiasis

**Complex PID**

- Cephalosporin or IgE-mediated penicillin allergy (quinolone resistant gonorrhea or antimicrobial susceptibility cannot be assessed)
- PID surgical complications (e.g., tubo-ovarian abscess)

**Complex herpes**

- Antiviral-resistant herpes infection
- Genital herpes contracted during third trimester of pregnancy
- Neonatal herpes

**Viral hepatitis**

- Hepatitis B infection
- Hepatitis C infection

**Complex warts**

- Cervical or intra-anal warts
- Atypical anogenital warts with high-grade squamous intraepithelial lesion on biopsy

**Cervical intraepithelial neoplasia or cervical cancer**

- Women with high- or low-grade squamous intraepithelial lesions on Pap smear

**Complex ectoparasitic infections**

- Crusted scabies in persons with HIV infection

**Sexual assault**

- HIV nPEP being considered
- STDs in children (if suspected possibility of sexual abuse)

**HIV infection**

- New diagnosis or establish link to care

**Abbreviations: **HIV = human immunodeficiency virus; IgE = immunoglobulin E; nPEP = nonoccupational postexposure prophylaxis; Pap = Papanicolaou; PID = pelvic inflammatory disease; PrEP = preexposure prophylaxis; STD = sexually transmitted disease.

## Future Directions

Research is needed to identify facilitators, barriers, and unintended consequences of implementing the recommendations for the specified STD or related clinical services in primary care and specialized STD care settings. High-priority research includes studies to quantify the impact of on-site or point-of-care services on patient compliance and health outcomes in various provider settings and patient populations.

## Conclusion

The recommendations in this report contribute to improved STD care by defining the STD or related clinical services that should be available in primary care and STD specialty care settings. Specialized STD care focuses on the delivery of timely, comprehensive, confidential, and culturally sensitive STD clinical services. Specialized STD care also preferably includes on-site immediate diagnosis (e.g., Gram stain or other stains for urethritis or stat nontreponemal serologic tests for syphilis) and on-site injectable antimicrobials to treat syphilis and gonorrhea. STD care in primary care settings might offer some of the same services as specialized STD care settings but, at a minimum, should encompass sexual history and risk assessment, screening, and treatment services. This guidance complements CDC’s *STD* *Guidelines* and outlines services for providing quality STD clinical services in primary care and STD specialty care settings. CDC will update *STD QCS* recommendations as more evidence become available and new practice standards are implemented.

## Footnotes

* The name of the Institute of Medicine was changed to the National Academy of Medicine, effective July 1, 2015 (http://www.nationalacademies.org/hmd/Global/News%20Announcements/IOM-to-become-NAM-Press-Release.aspx).
